# “Early earth” structural data from the Memve'ele area in the northwestern Congo Craton (Ntem complex-southwestern Cameroon)

**DOI:** 10.1016/j.dib.2020.106516

**Published:** 2020-11-10

**Authors:** Sylvestre M. NTOMBA, Dieudonné BISSO, Rufine C. MAGNEKOU TAKAMTE, François NDONG BIDZANG, Eric J. MESSI OTTOU, Joseph MVONDO ONDOA

**Affiliations:** a Centre for Geological and Mining Research, Institute of Geological and Mining Research, P.O. Box 333, Garoua, Cameroon; b Department of Earth Sciences, University of Yaoundé 1, P.O. Box 812, Yaoundé, Cameroon; c Ore Processing Laboratory (LTM), Institute of Geological and Mining Research, P.O. Box 4110, Yaoundé, Cameroon

**Keywords:** “Early earth” tectonics, Constriction, Transpression, Field investigation, Thin section observations, Memve'ele area, Ntem Complex, Cameroon

## Abstract

Structural data, meso‑ and micro-photographics were collected from Archean basement of the Memve'ele area (Ntem Complex, southwestern Cameroon). The analyses were acquired using field and laboratory investigations. Meso-photographics were obtained by a camera Canon *SX160 IS*, 16X digital zoom, HD 16.0 Mega pixels. Micro-photographics were carried out by electronic microscope Olympus *BX60* type with a camera and entire thin section picture scan. Structural data were acquired by a topochaix compass type and stereographic data were obtained by a stereonet program. The data presented in this paper are further interpreted and discussed in the Ntomba et al., 2020 [1].

## Specifications Table

**Table:** 

Subject	Earth sciences
Specific subject area	Structural geology
Type of data	Figures, plots and tables
How data were acquired	Field investigations, sampling, compass, camera, electronic microscope, scanning, ternary diagram and stereonet program.
Data format	Raw Analyzed
Parameters for data collection	Samples collected, structural features captured, and structural measurements taken in place on the field. Samples collected were orientated in the field to maintain the field sample orientation throughout the thin section. Thin section scanned.
Description of data collection	Structural features were captured at meso‑, micro-scales with camera and entire thin section scan. Strike, dip and trend, plunge measurements were considered to orientate foliations and lineations respectively; all these values were recorded and plotted on the ternary diagram; Obtained structural parameters with stereonet program are best poles, mean foliations and best lines; fold data capture allowed the delineation of fold axes and shortening trends.
Data source location	Memve'ele region (Ntem Complex, Southwestern Cameroon) as reported in [1].
Data accessibility	Data are available within this article and in the supplementary material.
Related research article	Sylvestre M. Ntomba, Dieudonné Bisso, Rufine C. Magnekou Takamte, François Ndong Bidzang, Eric J. Messi Ottou, Joseph Mvondo Ondoa. Crustal growth in the Mesoarchean plutonic belt from the Memve'ele area (Ntem Complex – Southwestern Cameroon): Evidence of “early earth” transpressional tectonics. *Journal of Structural Geology*. 141 (2020) 104,195 [1].

## Value of Data

• Foliation, folds, lineation and fold axes are structural patterns which represent a ductile deformation package for a comprehensive “early earth” tectonics of the Memve'ele region in the Ntem Complex (Southwestern Cameroon).
• Data enable recognition of “early earth” constrictional and transpressional regimes from the Memve'ele area.
• Data reveal significant variations in term of structural patterns orientation and can assist researchers to better understand the mechanisms of crustal growth during the lower Precambrian.
• Data contribute to highlight the characterization of structural patterns during the field investigation. This method is found as reliable tool to evaluate rapid crustal growth.
• The dataset contributes to efforts closing knowledge gap on structural geology in the Ntem Complex and could help to compare it with other cratons in the world.
• Data enable recognition of low and high temperature grades.

## 1 Data Description

**Fig. 1:**
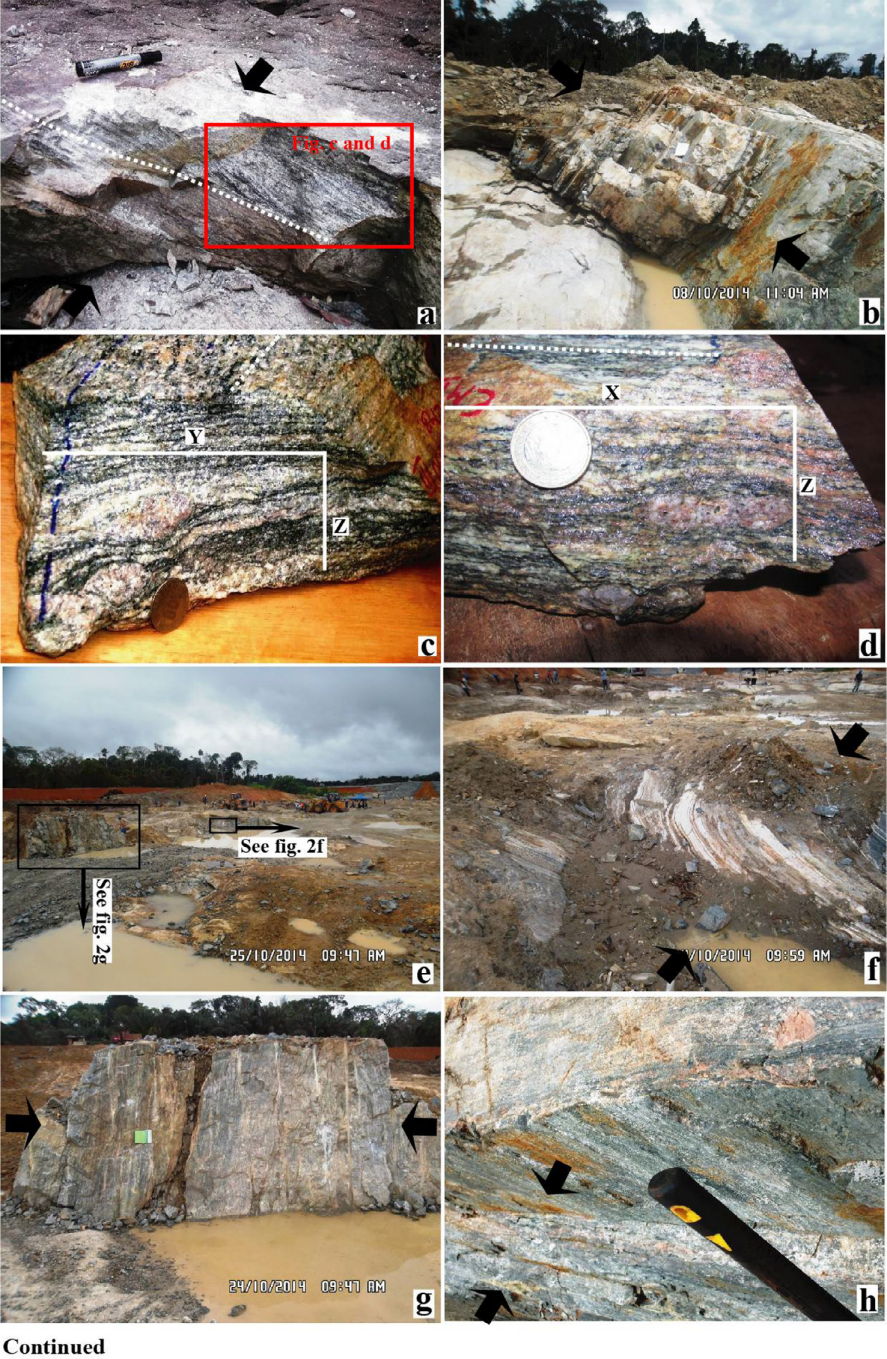

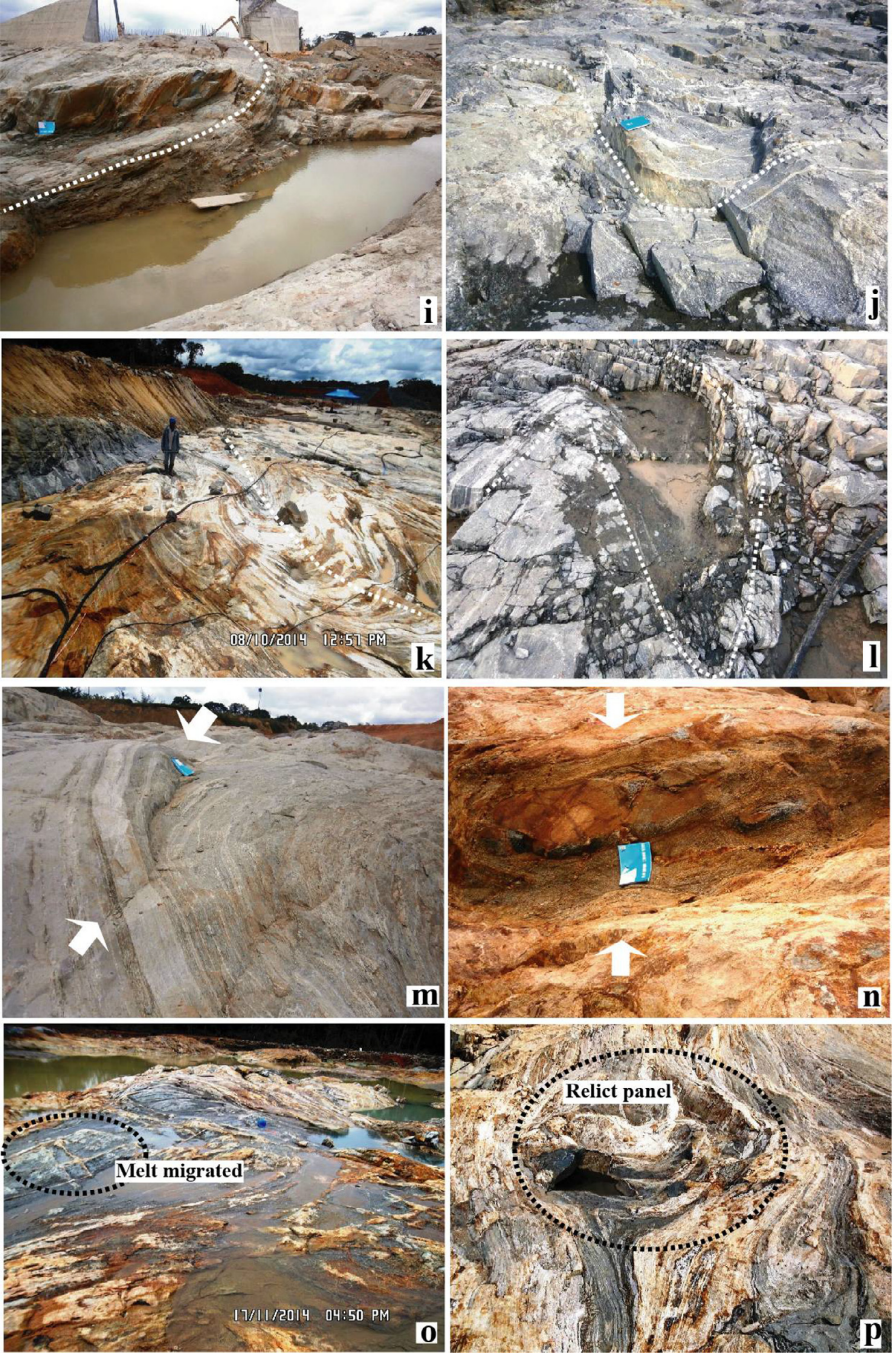
Field observations in the Memve'ele area.

**Fig. 2:**
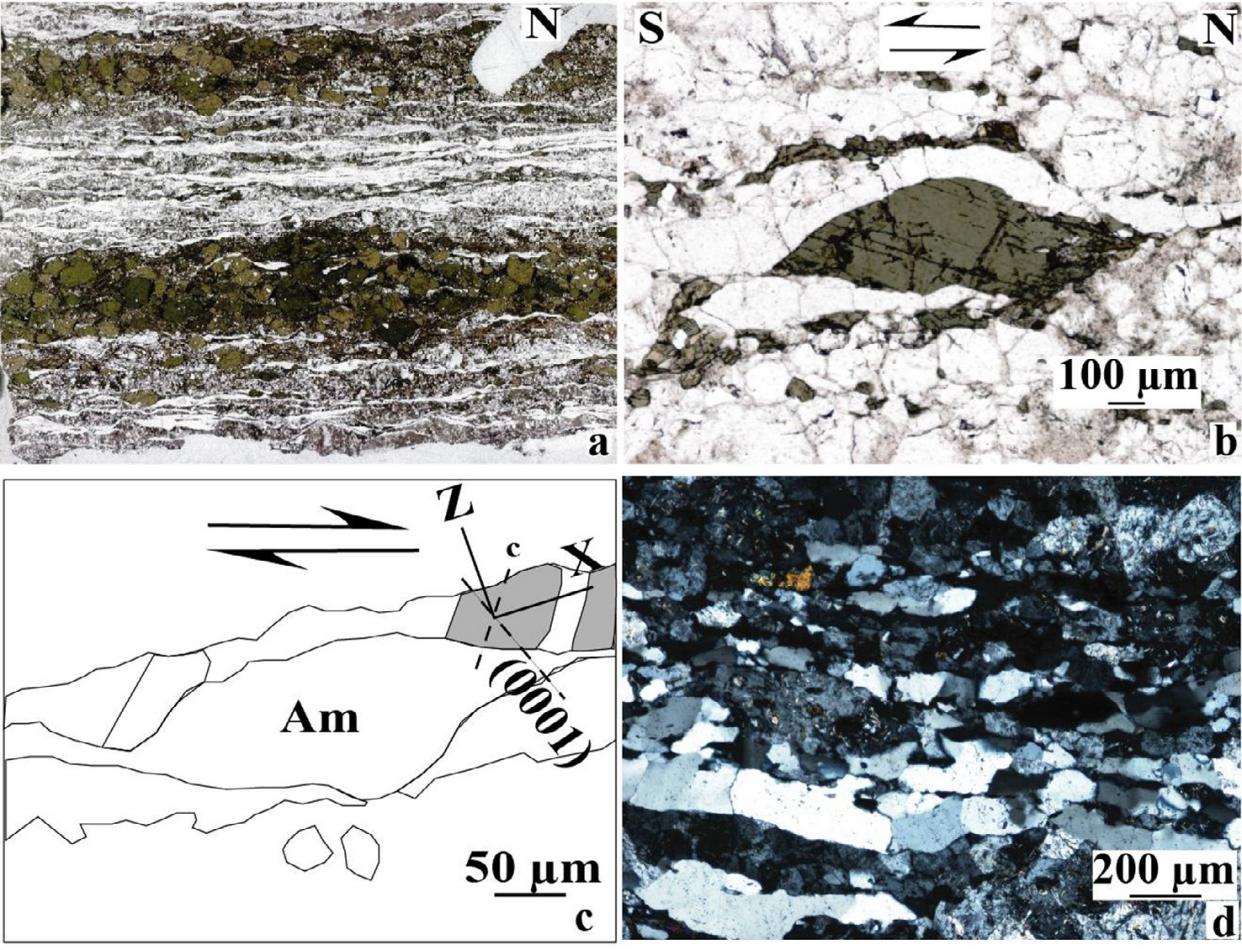
Photomicrographs showing some structural characters recorded by rocks from the Memve'ele shear zone (MSZ, [1]).

In this article we report structural data from the Memve'ele region in the Ntem Complex (Southwestern Cameroon). Data were obtained using different strategies such as field investigations, microscope observations and analytical methods. The most robust work on the field has allowed recording foliation and lineation measurements as reported in supplementary file and photo captures of structural patterns as shown in Fig. 1. Mineral behaviors have been captured, scanned and interpreted as shown in Fig. 2. Table 1 presents best poles, mean foliations, axe fold orientations and best lines obtained after calculations of the raw structural data (foliations and lineations) collected during field investigations and using stereonet program. The insight of tectonic regimes recorded in the Memve'ele region was obtained using ternary diagram of [3].

**Table 1:** Analyzed foliation and lineation data in the Memve'ele area (in red are axe fold traces).

Site numbers	Best poles	Mean foliations	Best lines	Site numbers	Best poles	Mean foliations	Best lines
1	134.54	N044.36NW N110.90		11	124.43	N034.47NNW	334/44
				12	198.28	N109.62	
2	165.53	N075.38NNW N105.20NNE	306/34 015/20	13	131.39	N041.51NW	006/39
3	137.50	N046.41NW	326/41	14	177.44	N087.46N	026/15
4	073.65 156.46	N164.26SW N066.44NW	246/25 350/37	16	186.47	N096.43 N N050.90	
				18	192.56	N102.34N	
5	156.47	N065.43NNW		19	164.43	N074.47N	
6	267.59	N177.32E N040.90		20	103.37	N013.53 W N015.90	067/90
7	174.50 336.75	N084.40 N N090.55 N N064.15S	006/36 040/39	21		N090.90	
				22	140.46	N051.45NW	358/37
				31	137.57	N048.33NW	350/25
8	162.49	N071.42NNW	352/38	32	122.51 084.22	N032.39NNW N174.68W	
9	164.42	N075.48NNW					
10	133.37	N043.53NW	001/42	33	164.45	N071.42NNW	

## 2 Experimental Design, Materials and Methods

### 2.1 Data and sample collections

Investigated sites belong to the Memve'ele region in the south Cameroon where structural measurements, lithologies sampling and structural patterns capture have been collected and done respectively (e.g. Fig. 1 and [1]). Mineral structural behaviors as reported in Fig. 2 and in entire thin section structural type (as shown in Fig. 2a) have been captured and scanned respectively in the GET laboratory (France).

### 2.2 Sample preparation

Lithology samples were previously marked by orientation traces during field investigations in order to preserve the orientation throughout the thin section process. Thin confections were carried out in the GET laboratory thin sections. For these preparations, thin sections were cut parallel to the aggregate lineation which represents (X) direction and normal to foliation plane (XY) [4], preserving orientation traces as shown in the Fig. 2a. Whole thin section was scanned in order to present an extensive deformation pattern as reported in Fig. 2a.

## 3 Geology

The regional fabrics pattern consists of shallow to steeply (∼10 ° to ∼85 °) dipping foliations with various directions; moderate to steeply (25 ° to 65 °) plunging and various strike parallel lineations (Table 1 and supplementary file).

The pictures captured show that the Memve'ele region displays a deformation pattern characterized by a shallowly dipping foliation (Fig. 1a, b and f), which is overprinted by a steeply dipping foliation (Fig. 1g and h) and a shallowly to steeply plunging and strike-parallel lineation (Fig. 1h). Pictures presenting foliation flattening planes, symmetric boudins and pintch and swell of feldspar in the YZ and XZ planes (Fig. 1b-c and h) were used to assess shortening directions as shown in Figs. 1a – b and f - h. Deformation on this region involves various shortening directions, forming folded foliation trace (Fig. 1i and j); various oriented trending folds including curved fold axis (Fig. 1k), superimposed folds (Fig. 1l), upright and sheath folds (Fig. 1m and n). Insight of partial melting in this region is shown in Figs. 1o and p.

Fig. 2a displays microstructures of symmetric amphibole bands in the alternation of quartzo - plagioclassic ribbons parallel to stretching direction. Kinematic patterns are rarely seen, but locally amphibole stair stepping (Fig. 2b) and c-axis quartz fabric as shown in interpretative sketch picture (Fig. 2c) indicate displacement directions. Fig. 2d displays quartz ribbon with curved sub-grain boundaries or an activation of “a” gliding system.

**Fig. 3:**
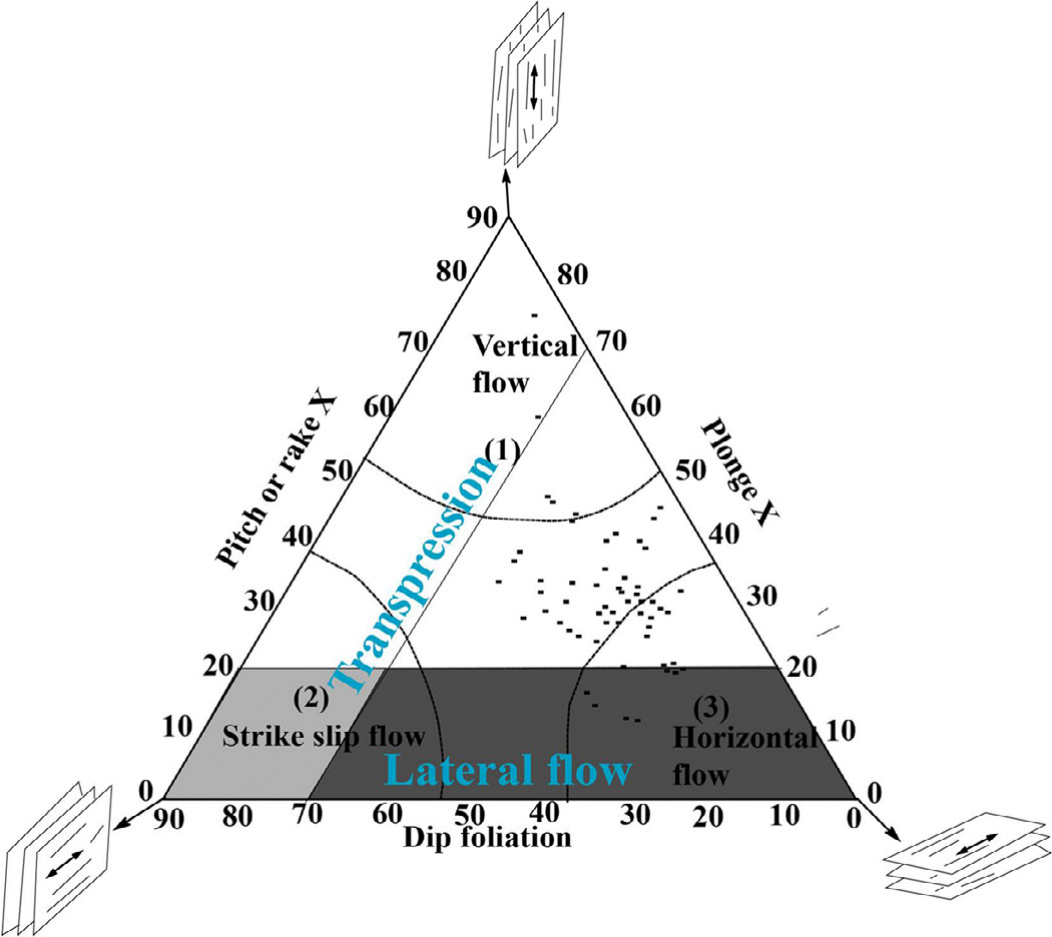
Structural pattern data (foliation and lineation) plotted on dip-pitch-plunge triangular diagram of [2] (modified after a diagram concept of [3]). Noted that steep of both foliation and lineation data are not shown.

Foliation and lineation measurements recorded during field investigations in the shear zone have been plotted in Fig. 3 in order to characterize tectonic regimes.

## CRediT Author Statement

**Sylvestre M. Ntomba:** Conceptualization, Methodology, Data curation, Project administration. **Dieudonné Bisso:** Visualization, Investigation. **Rufine C. Magnekou Takamte:** Writing - original draft. **François Ndong Bidzang:** Formal analysis. **Eric J. Messi Ottou:** Writing - review & editing. **Joseph Mvondo Ondoa:** Supervision.

## Declaration of Competing Interest

The authors declare that they have no known competing financial interests or personal relationships that could have appeared to influence the work reported in this paper.

## Appendix Supplementary Materials

Image, application 1
