# Supplementary material for: “Early earth” structural data from the Memve'ele area in the northwestern Congo Craton (Ntem complex-southwestern Cameroon)
Source: Data Brief. 2020 Nov 10;33:106516. doi: 10.1016/j.dib.2020.106516 (PMC7689373; doi:10.1016/j.dib.2020.106516)
Supplement: Supplementary file 1 [file mmc1.docx]

**Supplementary files:**

**Supplementary file:** Raw foliation and lineation data.

**FOLIATION DATA**

1. **Out of dam site**

| **N°** | **Strike** | **Dip** | **Dip quad** | **N°** | **Strike** | **Dip** | **Dip quad** | **N°** | **Strike** | **Dip** | **Dip**  **quad** |
| --- | --- | --- | --- | --- | --- | --- | --- | --- | --- | --- | --- |

**1** 220,0 25,0 W

**2** 234,0 23,0 N

**3** 230,0 33,0 N

**4** 244,0 24,0 N

**5** 250,0 30,0 N

**6** 240,0 40,0 N

**7** 234,0 41,0 N

**8** 208,0 43,0 W

**9** 220,0 38,0 W

**10** 210,0 40,0 W

**11** 217,0 30,0 W

**12** 230,0 25,0 N

**13** 218,0 38,0 W

**14** 222,0 27,0 W

**15** 217,0 32,0 W

**16** 223,0 31,0 W

**17** 215,0 42,0 W

**18** 217,0 40,0 W

**19** 220,0 38,0 W

**20** 216,0 43,0 W

**21** 229,0 37,0 N

**22** 204,0 53,0 W

**23** 202,0 25,0 W

**24** 258,0 40,0 N

**25** 268,0 43,0 N

**26** 235,0 55,0 N

**27** 235,0 46,0 N

**28** 244,0 17,0 N

**29** 238,0 36,0 N

**30** 236,0 32,0 N

**31** 243,0 45,0 N

**32** 220,0 35,0 W

**33** 230,0 36,0 N

**34** 218,0 34,0 W

**35** 227,0 38,0 N

**36** 242,0 40,0 N

**37** 232,0 42,0 N

**38** 235,0 44,0 N

**39** 216,0 45,0 W

**40** 250,0 62,0 N

**41** 244,0 41,0 N

**42** 242,0 40,0 N

**43** 242,0 45,0 N

**44** 234,0 42,0 N

**45** 238,0 40,0 N

**46** 280,0 48,0 N

**47** 250,0 40,0 N

**48** 272,0 38,0 N

**49** 258,0 34,0 N

**50** 272,0 46,0 N

**51** 250,0 42,0 N

**52** 238,0 45,0 N

**53** 240,0 46,0 N

**54** 250,0 42,0 N

**55** 238,0 45,0 N

**56** 240,0 46,0 N

**57** 268,0 32,0 N

**58** 270,0 38,0 N

**59** 272,0 35,0 N

**60** 262,0 38,0 N

**61** 272,0 37,0 N

**62** 258,0 34,0 N

**63** 270,0 40,0 N

**64** 272,0 35,0 N

**65** 262,0 38,0 N

**66** 258,0 32,0 N

**67** 262,0 36,0 N

**68** 260,0 38,0 N

**69** 272,0 30,0 N

**70** 282,0 35,0 N

**71** 298,0 32,0 N

**72** 278,0 44,0 N

**73** 272,0 34,0 N

**74** 248,0 40,0 N

**75** 260,0 32,0 N

**76** 258,0 36,0 N

**77** 272,0 32,0 N

**78** 250,0 33,0 N

**79** 290,0 35,0 N

**80** 272,0 38,0 N

**81** 212,0 35,0 W

**82** 212,0 26,0 W

**83** 228,0 34,0 N

**84** 230,0 48,0 N

**85** 238,0 52,0 N

**86** 208,0 45,0 W

**87** 222,0 48,0 W

**88** 232,0 45,0 N

**89** 242,0 42,0 N

**90** 240,0 48,0 N

**91** 240,0 44,0 N

**92** 230,0 48,0 N

**93** 241,0 50,0 N

**94** 242,0 50,0 N

**95** 258,0 60,0 N

**96** 220,0 48,0 W

**97** 210,0 62,0 W

**98** 228,0 50,0 N

**99** 230,0 42,0 N

**100** 228,0 46,0 N

**101** 256,0 42,0 N

**102** 252,0 36,0 N

**103** 272,0 32,0 N

**104** 250,0 33,0 N

**105** 290,0 35,0 N

**106** 272,0 38,0 N

**107** 212,0 35,0 W

**108** 212,0 26,0 W

**109** 228,0 34,0 N

**110** 230,0 48,0 N

**111** 238,0 52,0 N

**112** 208,0 45,0 W

**113** 222,0 42,0 W

**114** 232,0 45,0 N

**115** 242,0 42,0 N

**116** 240,0 48,0 N

**117** 240,0 44,0 N

**118** 230,0 48,0 N

**119** 241,0 50,0 N

**120** 242,0 50,0 N

**121** 258,0 60,0 N

**122** 220,0 48,0 W

**123** 210,0 62,0 W

**124** 228,0 50,0 N

**125** 230,0 42,0 N

**126** 228,0 46,0 N

**127** 256,0 42,0 N

**128** 252,0 36,0 N

**129** 254,0 46,0 N

**130** 232,0 38,0 N

**131** 234,0 54,0 N

**132** 254,0 46,0 N

**133** 268,0 55,0 N

**134** 270,0 52,0 N

**135** 266,0 58,0 N

**136** 270,0 48,0 N

**137** 282,0 42,0 N

**138** 262,0 42,0 N

**139** 280,0 62,0 N

**140** 270,0 48,0 N

**141** 278,0 52,0 N

**142** 282,0 54,0 N

**143** 278,0 54,0 N

**144** 262,0 50,0 N

**145** 252,0 44,0 N

**146** 232,0 54,0 N

**147** 242,0 54,0 N

**148** 212,0 48,0 W

**149** 210,0 55,0 W

**150** 248,0 45,0 N

**151** 250,0 48,0 N

**152** 282,0 54,0 N

**153** 270,0 50,0 N

**154** 278,0 48,0 N

**155** 270,0 46,0 N

**156** 270,0 54,0 N

**157** 250,0 40,0 N

**158** 282,0 54,0 N

**159** 270,0 50,0 N

**160** 278,0 48,0 N

**161** 270,0 46,0 N

**162** 270,0 54,0 N

**163** 239,0 56,0 N

**164** 242,0 60,0 N

**165** 238,0 58,0 N

**166** 208,0 38,0 W

**167** 210,0 44,0 W

**168** 220,0 45,0 W

**169** 232,0 62,0 N

**170** 232,0 64,0 N

**171** 222,0 58,0 W

**172** 218,0 57,0 W

**173** 230,0 54,0 N

**174** 232,0 56,0 N

**175** 222,0 46,0 W

**176** 228,0 52,0 N

**177** 226,0 42,0 N

**178** 218,0 48,0 W

**179** 222,0 62,0 W

**180** 228,0 60,0 N

**181** 224,0 52,0 W

**182** 230,0 52,0 N

**183** 208,0 60,0 W

**184** 334,0 49,0 E

**185** 210,0 50,0 W

**186** 242,0 34,0 N

**187** 230,0 34,0 N

**188** 212,0 38,0 W

**189** 218,0 40,0 W

**190** 230,0 38,0 N

**191** 230,0 42,0 N

**192** 212,0 38,0 W

**193** 222,0 32,0 W

**194** 222,0 28,0 W

**195** 262,0 36,0 N

**196** 242,0 42,0 N

**197** 222,0 38,0 W

**198** 224,0 40,0 W

**199** 238,0 35,0 N

**200** 240,0 28,0 N

**201** 200,0 38,0 W

**202** 228,0 35,0 N

**203** 258,0 44,0 N

**204** 240,0 40,0 N

**205** 250,0 46,0 N

**206** 252,0 42,0 N

**207** 252,0 39,0 N

**208** 258,0 44,0 N

**209** 240,0 41,0 N

**210** 250,0 43,0 N

**211** 244,0 34,0 N

**212** 244,0 39,0 N

**213** 240,0 39,0 N

**214** 232,0 38,0 N

**215** 239,0 40,0 N

**216** 238,0 52,0 N

**217** 258,0 36,0 N

**218** 214,0 38,0 W

**219** 216,0 48,0 W

**220** 245,0 36,0 N

**221** 242,0 42,0 N

**222** 288,0 38,0 N

**223** 230,0 35,0 N

**224** 280,0 46,0 N

**225** 295,0 48,0 N

**226** 298,0 48,0 N

**227** 278,0 44,0 N

**228** 298,0 42,0 N

**229** 284,0 38,0 N

**230** 300,0 32,0 N

**231** 250,0 55,0 N

**232** 222,0 54,0 W

**233** 238,0 42,0 N

**234** 270,0 45,0 N

**235** 268,0 46,0 N

**236** 292,0 50,0 N

**237** 310,0 51,0 N

**238** 310,0 52,0 N

**239** 285,0 54,0 N

**240** 270,0 45,0 N

**241** 260,0 30,0 N

**242** 268,0 40,0 N

**243** 280,0 47,0 N

**244** 288,0 35,0 N

**245** 260,0 38,0 N

**246** 280,0 30,0 N

**247** 250,0 29,0 N

**248** 252,0 22,0 N

**249** 298,0 34,0 N

**250** 212,0 55,0 W

**251** 210,0 48,0 W

**252** 212,0 52,0 W

**253** 210,0 46,0 W

**254** 218,0 55,0 W

**255** 210,0 46,0 W

**256** 218,0 55,0 W

**257** 212,0 53,0 W

**258** 210,0 53,0 W

**259** 208,0 48,0 W

**260** 222,0 54,0 W

**261** 202,0 42,0 W

**262** 218,0 40,0 W

**263** 214,0 42,0 W

**264** 222,0 38,0 W

**265** 220,0 38,0 W

**266** 210,0 40,0 W

**267** 212,0 46,0 W

**268** 212,0 50,0 W

**269** 222,0 43,0 W

**270** 230,0 45,0 N

**271** 222,0 46,0 W

**272** 228,0 45,0 N

**273** 208,0 48,0 W

**274** 208,0 46,0 W

**275** 220,0 38,0 W

**276** 210,0 44,0 W

**277** 220,0 35,0 W

**278** 198,0 51,0 W

**279** 218,0 42,0 W

**280** 200,0 45,0 W

**281** 210,0 44,0 W

**282** 206,0 46,0 W

**283** 200,0 45,0 W

**284** 210,0 44,0 W

**285** 206,0 46,0 W

**286** 228,0 50,0 N

**287** 228,0 48,0 N

**288** 232,0 46,0 N

**289** 222,0 46,0 W

**290** 240,0 54,0 N

**291** 220,0 45,0 W

**292** 218,0 46,0 W

**293** 200,0 46,0 W

**294** 220,0 44,0 W

**295** 200,0 45,0 W

**296** 208,0 48,0 W

**297** 212,0 52,0 W

**298** 240,0 55,0 N

**299** 212,0 50,0 W

**300** 218,0 48,0 W

**301** 210,0 50,0 W

**302** 212,0 58,0 W

**303** 212,0 52,0 W

**304** 208,0 50,0 W

**305** 222,0 70,0 W

**306** 228,0 50,0 N

**307** 220,0 38,0 W

**308** 230,0 40,0 N

**309** 207,0 29,0 W

**310** 218,0 14,0 W

**311** 228,0 41,0 N

**312** 220,0 35,0 W

**313** 226,0 43,0 N

**314** 236,0 35,0 N

**315** 232,0 38,0 N

**316** 232,0 36,0 N

**317** 244,0 24,0 N

**318** 235,0 24,0 N

**319** 216,0 15,0 W

**320** 213,0 23,0 W

**321** 217,0 27,0 W

**322** 245,0 28,0 N

**323** 236,0 28,0 N

**324** 236,0 30,0 N

**325** 243,0 28,0 N

**326** 228,0 39,0 N

**327** 242,0 24,0 N

**328** 230,0 28,0 N

**329** 228,0 48,0 N

**330** 224,0 34,0 W

**331** 222,0 36,0 W

**332** 224,0 39,0 W

**333** 227,0 42,0 N

**334** 218,0 45,0 W

**335** 224,0 48,0 W

**336** 225,0 50,0 W

**337** 228,0 26,0 N

**338** 210,0 15,0 W

**339** 212,0 47,0 W

**340** 210,0 58,0 W

**341** 206,0 50,0 W

**342** 210,0 59,0 W

**343** 214,0 54,0 W

**344** 205,0 63,0 W

**345** 264,0 37,0 N

**346** 210,0 59,0 W

**347** 212,0 57,0 W

**348** 210,0 50,0 W

**349** 220,0 53,0 W

**350** 208,0 43,0 W

**351** 240,0 35,0 N

**352** 238,0 44,0 N

**353** 230,0 42,0 N

**354** 240,0 42,0 N

**355** 232,0 40,0 N

**356** 238,0 40,0 N

**357** 242,0 42,0 N

**358** 230,0 39,0 N

**359** 242,0 44,0 N

**360** 280,0 35,0 N

**361** 248,0 40,0 N

**362** 258,0 40,0 N

**363** 260,0 42,0 N

**364** 258,0 38,0 N

**365** 250,0 42,0 N

**366** 240,0 51,0 N

**367** 238,0 47,0 N

**368** 242,0 45,0 N

**369** 241,0 55,0 N

**370** 242,0 53,0 N

**371** 233,0 47,0 N

**372** 243,0 52,0 N

**373** 248,0 45,0 N

**374** 252,0 45,0 N

**375** 246,0 46,0 N

**376** 254,0 44,0 N

**377** 240,0 51,0 N

**378** 231,0 47,0 N

**379** 250,0 68,0 N

**380** 270,0 40,0 N

**381** 280,0 42,0 N

**382** 260,0 43,0 N

**383** 240,0 44,0 N

**384** 212,0 52,0 W

**385** 222,0 58,0 W

**386** 252,0 45,0 N

**387** 238,0 52,0 N

**388** 260,0 56,0 N

**389** 258,0 50,0 N

**390** 258,0 68,0 N

**391** 210,0 64,0 W

**392** 238,0 50,0 N

**393** 232,0 48,0 N

**394** 208,0 52,0 W

**395** 210,0 55,0 W

**396** 240,0 52,0 N

**397** 208,0 46,0 W

**398** 222,0 52,0 W

**399** 228,0 46,0 N

**400** 208,0 54,0 W

**401** 258,0 50,0 N

**402** 230,0 48,0 N

**403** 222,0 52,0 W

**404** 218,0 50,0 W

**405** 232,0 42,0 N

**406** 212,0 36,0 W

**407** 183,0 58,0 W

**408** 182,0 46,0 W

**409** 184,0 44,0 W

**410** 185,0 50,0 W

**411** 186,0 56,0 W

**412** 210,0 42,0 W

**413** 212,0 46,0 W

**414** 222,0 68,0 W

**415** 202,0 60,0 W

**416** 232,0 64,0 N

**417** 232,0 66,0 N

**418** 222,0 64,0 W

**419** 242,0 48,0 N

**420** 232,0 46,0 N

**421** 238,0 42,0 N

**422** 220,0 46,0 W

**423** 202,0 56,0 W

**424** 208,0 52,0 W

**425** 212,0 44,0 W

**426** 238,0 52,0 N

**427** 244,0 50,0 N

**428** 240,0 55,0 N

**429** 218,0 50,0 W

**430** 228,0 60,0 N

**431** 222,0 50,0 W

**432** 222,0 48,0 W

**433** 238,0 52,0 N

**434** 244,0 50,0 N

**435** 240,0 55,0 N

**436** 210,0 50,0 W

**437** 208,0 48,0 W

**438** 192,0 52,0 W

**439** 208,0 55,0 W

**440** 216,0 54,0 W

**441** 228,0 60,0 N

**442** 238,0 54,0 N

**443** 232,0 58,0 N

**444** 222,0 55,0 W

**445** 200,0 56,0 W

**446** 200,0 52,0 W

**447** 200,0 62,0 W

**448** 228,0 52,0 N

**449** 208,0 55,0 W

**450** 238,0 60,0 N

**451** 272,0 38,0 N

**452** 252,0 26,0 N

**453** 258,0 34,0 N

**454** 292,0 32,0 N

**455** 298,0 58,0 N

**456** 268,0 52,0 N

**457** 262,0 54,0 N

**458** 252,0 48,0 N

**459** 270,0 52,0 N

**460** 268,0 60,0 N

**461** 242,0 46,0 N

**462** 244,0 54,0 N

**463** 272,0 45,0 N

**464** 258,0 38,0 N

**465** 262,0 46,0 N

**466** 282,0 46,0 N

**467** 292,0 42,0 N

**468** 272,0 56,0 N

**469** 288,0 52,0 N

**470** 258,0 48,0 N

**471** 310,0 48,0 N

**472** 298,0 40,0 N

**473** 282,0 36,0 N

**474** 222,0 46,0 W

**475** 282,0 34,0 N

**476** 202,0 62,0 W

**477** 208,0 42,0 W

**478** 222,0 66,0 W

**479** 180,0 58,0 W

**480** 182,0 56,0 W

**481** 190,0 62,0 W

**482** 150,0 64,0 W

**483** 194,0 50,0 W

**484** 192,0 42,0 W

**485** 194,0 56,0 W

**486** 188,0 52,0 W

**487** 188,0 48,0 W

**488** 197,0 46,0 W

**489** 192,0 55,0 W

**490** 184,0 61,0 W

**491** 188,0 63,0 W

**492** 192,0 50,0 W

**493** 204,0 47,0 W

**494** 202,0 47,0 W

**495** 205,0 45,0 W

**496** 195,0 65,0 W

**497** 204,0 54,0 W

**498** 194,0 48,0 W

**499** 200,0 40,0 W

**500** 190,0 42,0 W

**501** 197,0 64,0 W

**502** 180,0 65,0 W

**503** 140,0 34,0 W

**504** 174,0 30,0 W

**505** 238,0 45,0 N

**506** 238,0 55,0 N

**507** 170,0 60,0 W

**508** 200,0 50,0 W

**509** 190,0 40,0 W

**510** 180,0 32,0 W

**511** 186,0 34,0 W

**512** 215,0 40,0 W

**513** 203,0 37,0 W

**514** 245,0 41,0 N

**515** 258,0 42,0 N

**516** 238,0 47,0 N

**517** 184,0 59,0 W

**518** 196,0 68,0 W

**519** 198,0 54,0 W

**520** 195,0 50,0 W

**521** 202,0 43,0 W

**522** 188,0 53,0 W

**523** 190,0 50,0 W

**524** 180,0 55,0 W

**525** 195,0 58,0 W

**526** 192,0 52,0 W

**527** 183,0 45,0 W

**528** 180,0 51,0 W

**529** 240,0 40,0 N

**530** 198,0 40,0 W

**531** 257,0 50,0 N

**532** 195,0 58,0 W

**533** 240,0 52,0 N

**534** 242,0 47,0 N

**535** 232,0 42,0 N

**536** 222,0 54,0 W

**537** 238,0 46,0 N

**538** 222,0 64,0 W

**539** 222,0 38,0 W

**540** 230,0 36,0 N

**541** 218,0 42,0 W

**542** 248,0 44,0 N

**543** 238,0 42,0 N

**544** 248,0 36,0 N

**545** 218,0 52,0 W

**546** 212,0 55,0 W

**547** 210,0 48,0 W

**548** 212,0 52,0 W

**549** 210,0 46,0 W **550** 218,0 55,0 W

**551** 212,0 53,0 W

**552** 210,0 53,0 W

**553** 208,0 48,0 W

**554** 222,0 54,0 W

**555** 202,0 42,0 W

**556** 218,0 40,0 W

**557** 214,0 42,0 W

**558** 222,0 38,0 W

**559** 220,0 38,0 W

**560** 210,0 40,0 W

**561** 212,0 46,0 W

**562** 212,0 50,0 W

**563** 222,0 43,0 W

**564** 230,0 45,0 N

**565** 222,0 46,0 W

**566** 228,0 45,0 N

**567** 208,0 48,0 W

**568** 208,0 46,0 W

**569** 220,0 38,0 W

**570** 200,0 38,0 W

**571** 210,0 44,0 W

**572** 220,0 38,0 W

**573** 198,0 51,0 W

**574** 218,0 42,0 W

**575** 200,0 45,0 W

**576** 210,0 44,0 W

**577** 206,0 46,0 W

**578** 200,0 45,0 W

**579** 210,0 44,0 W

**580** 206,0 46,0 W

**581** 228,0 50,0 N

**582** 228,0 48,0 N

**583** 232,0 46,0 N

**584** 222,0 46,0 W

**585** 240,0 54,0 N

**586** 220,0 45,0 W

**587** 218,0 46,0 W

**588** 200,0 46,0 W

**589** 220,0 44,0 W

**590** 200,0 45,0 W

**591** 208,0 48,0 W

**592** 212,0 52,0 W

**593** 240,0 55,0 N

**594** 212,0 50,0 W

**595** 218,0 48,0 W

**596** 210,0 50,0 W

**597** 212,0 58,0 W

**598** 212,0 53,0 W

**599** 208,0 50,0 W

**600** 222,0 70,0 W

**601** 228,0 50,0 N

**602** 220,0 38,0 W

**603** 230,0 40,0 N

**604** 200,0 52,0 W

**605** 202,0 47,0 W

**606** 210,0 58,0 W

**607** 206,0 50,0 W

**608** 210,0 59,0 W

**609** 214,0 54,0 W

**610** 208,0 34,0 W

**611** 205,0 63,0 W

**612** 264,0 37,0 N

**613** 210,0 59,0 W

**614** 212,0 57,0 W

**615** 210,0 50,0 W

**616** 220,0 53,0 W

**617** 208,0 43,0 W

**618** 165,0 68,0 W

**619** 163,0 78,0 W

**620** 160,0 80,0 W

**621** 173,0 72,0 W

**622** 162,0 74,0 W

**623** 170,0 80,0 W

**624** 170,0 78,0 W

**625** 185,0 79,0 W

**626** 184,0 72,0 W

**627** 182,0 53,0 W

**628** 180,0 66,0 W

**629** 180,0 84,0 W

**630** 185,0 61,0 W

**631** 190,0 59,0 W

**632** 170,0 54,0 W

**633** 169,0 58,0 W

**634** 174,0 50,0 W

**LINEATIONS**

| **N°** | **Trend** | **Plunge** |
| --- | --- | --- |

**1** 348,0 38,0

**2** 358,0 54,0

**3** 330,0 48,0

**4** 333,0 60,0

**5** 360,0 25,0

**6** 330,0 59,0

**7** 330,0 50,0

**8** 329,0 50,0

**9** 340,0 46,0

**10** 340,0 42,0

**11** 322,0 42,0

**12** 330,0 38,0

**13** 331,0 36,0

**14** 338,0 33,0

**15** 312,0 38,0

**16** 330,0 36,0

**17** 330,0 37,0

**18** 320,0 40,0

**b- Within of dam site**

| **N°** | **Strike** | **Dip** | **Dip quad** |
| --- | --- | --- | --- |

**1** 230 68 NW

**2** 222 64 NW

**3** 236 70 NW

**4** 222 70 NW

**5** 228 52 NW

**6** 216 64 NW

**7** 216 68 NW

**8** 218 64 NW

**9** 200 68 NW

**10** 228 65 NW

**11** 234 62 NW

**12** 247 66 NW

**13** 248 58 NNW

**14** 220 62 NW

**15** 204 70 W

**16** 240 62 NW

**17** 202 54 W

**18** 220 58 NW

**19** 222 62 NW

**20** 270 70 N

**21** 260 50 N

**22** 286 50 N

**23** 220 32 NW

**24** 205 50 NW

**25** 205 58 NW

**26** 280 40 N

**27** 234 38 NW

**28** 206 48 W

**29** 225 42 NW

**30** 260 68 N

**31** 280 64 N

**32** 296 72 N

**33** 314 70 NE

**34** 278 43 N

**35** 206 48 W

**36** 225 42 NW

**37** 260 68 N

**38** 280 64 N

**39** 296 72 N

**40** 314 70 NE

**41** 278 43 N

**42** 182 70 N

**43** 300 70 N

**44** 292 74 N

**45** 260 24 N

**46** 260 68 N

**47** 280 70 N

**48** 218 48 NW

**49** 280 40 N

**50** 164 30 WSW

**51** 167 48 W

**52** 164 50 W

**53** 258 42 NW

**54** 266 40 N

**55** 138 52 SW

**56** 292 46 N

**57** 248 52 NW

**58** 138 90

**59** 062 90

**60** 144 90

**61** 159 90

**62** 152 90

**63** 244 42 NW

**64** 238 42 NW

**65** 172 40 W

**66** 310 80 NE

**67** 248 42 NW

**68** 265 50 N

**69** 240 48 NNW

**70** 262 48 N

**71** 260 48 N

**72** 236 44 NW

**73** 238 50 NW

**74** 248 46 NW

**75** 236 42 NW

**76** 216 52 NW

**77** 164 64 W

**78** 194 50 W

**79** 158 68 WSW

**80** 276 58 N

**81** 258 68 NW

**82** 270 70 N

**83** 220 32 NW

**84** 286 50 N

**85** 260 50 N

**86** 252 44 NW

**87** 206 54 W

**88** 246 48 NNW

**89** 196 88 W

**90** 016 90

**91** 220 62 NW

**92** 280 62 NW

**93** 280 60 N

**94** 312 82 NE

**95** 130 84 SW

**96** 153 52 WSW

**97** 320 80 NE

**98** 164 62 W

**99** 182 42 W

**100** 150 78 W

**101** 202 82 WNW

**102** 192 50 W

**103** 144 50 SW

**104** 220 68 NW

**105** 284 58 N

**106** 244 70 N

**107** 222 50 NW

**108** 244 56 NNW

**109** 238 58 NW

**110** 246 72 NW

**111** 278 70 N

**112** 282 68 N

**113** 250 52 NNW

**114** 220 52 NW

**115** 198 54 W

**116** 212 38 NW

**117** 260 42 N

**118** 270 52 N

**119** 280 50 N

**120** 163 50 W

**121** 276 70 N

**122** 144 52 SW

**123** 198 44 W

**124** 228 38 NW

**125** 302 70 NNE

**126** 300 62 NNE

**127** 300 62 NNE

**128** 250 40 NW

**129** 230 42 NW

**130** 240 50 NNW

**131** 264 70 N

**132** 270 72 N

**133** 280 70 N

**134** 278 68 N

**135** 218 70 NW

**136** 278 46 N

**137** 300 80 N

**138** 152 50 WSW

**139** 218 52 NW

**140** 160 52 WSW

**141** 054 90

**142** 166 54 WSW

**143** 240 52 NNW

**144** 194 62 W

**145** 252 58 NNW

**146** 300 44 NNE

**147** 260 54 N

**148** 152 58 SW

**149** 138 60 SW

**150** 66 46 SE

**151** 22 80 E

**152** 022 90

**153** 244 48 NW

**154** 210 38 NW

**155** 146 60 SW

**156** 140 54 SW

**157** 136 62 SW

**158** 138 50 SW

**159** 240 34 NW

**160** 267 52 N

**161** 90 60 S

**162** 152 52 SW

**163** 303 64 NE

**164** 250 80 N

**165** 120 90

**166** 260 72 N

**167** 120 86 S

**168** 115 70 S

**169** 112 72 S

**170** 184 46 W

**171** 200 43 W

**172** 192 42 W

**173** 154 52 W

**174** 202 38 W

**175** 228 46 NW

**176** 148 62 SW

**177** 132 68 SW

**178** 96 64 S

**179** 140 62 SW

**180** 58 58 SE

**181** 130 58 SW

**182** 204 40 W

**183** 256 46 NW

**184** 282 66 N

**185** 218 42 NW

**186** 194 38 W

**187** 164 38 W

**188** 270 40 N

**189** 286 78 N

**190** 258 44 NNW

**191** 238 38 NW

**192** 193 42 W

**193** 198 40 W

**194** 208 42 W

**195** 250 38 NNW

**196** 208 36 W

**197** 163 64 W

**198** 160 60 W

**199** 160 58 W

**200** 180 52 W

**201** 164 56 W

**202** 160 52 W

**203** 216 44 NW

**204** 247 54 NW

**205** 262 67 N

**206** 240 32 NW

**207** 086 90

**208** 86 78 S

**209** 120 78 SSW

**210** 124 62 SSW

**211** 124 68 SSW

**212** 134 68 SW

**213** 178 50W

**214** 180 52 W

**215** 160 53 W

**216** 160 66 W

**217** 174 42 W

**218** 170 40 W

**219** 162 38 W

**220** 172 48 W

**221** 180 46 W

**222** 182 46 W

**223** 148 62 SW

**224** 132 62 SW

**225** 240 42 NW

**226** 232 40 NW

**227** 236 32 NW

**228** 230 40 NW

**229** 323 60 NE

**230** 310 80 NE

**231** 248 42 NW

**232** 306 48 NE

**233** 090 90

**234** 116 72 S

**235** 130 58 SW

**236** 152 52 WSW

**237** 174 42 W

**238** 196 34 W

**239** 148 56 SW

**240** 132 58 SW

**241** 284 60 N

**242** 94 70 S

**243** 258 72 N

**244** 182 50 W

**245** 180 38 W

**246** 110 90

**247** 120 66 SW

**248** 138 62 SW

**249** 140 90

**250** 274 58 N

**251** 144 72 SW

**252** 212 52 WNW

**253** 242 62 NNW

**254** 270 64 N

**255** 266 66 N

**256** 180 48 W

**257** 172 50 W

**258** 170 62 W

**259** 194 34 W

**260** 258 54 NNW

**261** 264 60 N

**262** 270 44 N

**263** 283 62 N

**264** 290 53 NNE

**265** 304 56 NE

**266** 322 62 NE

**267** 216 42 NW

**268** 280 40 N

**269** 296 34 NNE

**270** 270 38 N

**271** 224 54 W

**272** 234 49 W

**273** 226 54 W

**274** 228 46 W

**275** 222 58 W

**276** 235 74 W

**277** 234 72 W

**278** 234 75 W

**279** 210 79 W

**280** 215 75 W

**281** 248 68 W

**282** 250 64 N

**283** 244 60 W

**284** 212 78 W

**285** 242 72 N

**286** 240 76 N

**287** 242 64 N

**288** 258 52 N

**289** 240 56 N

**290** 238 58 N

**291** 248 52 N

**292** 264 60 N

**293** 234 50 N

**294** 246 42 N

**295** 250 54 N

**296** 248 50 N

**297** 260 49 N

**298** 242 66 N

**299** 250 50 N

**300** 255 56 N

**301** 242 53 N

**302** 248 52 N

**303** 250 46 N

**304** 234 50 N

**305** 292 72 N

**306** 290 60 N

**307** 300 62 N

**308** 308 53 N

**309** 300 62 N

**310** 295 48 N

**311** 290 49 N

**312** 278 53 N

**313** 274 50 N

**314** 270 58 N

**315** 274 42 N

**316** 294 47 N

**317** 295 56 N

**318** 282 62 N

**319** 305 45 N

**320** 298 48 N

**321** 295 42 N

**322** 285 45 N

**323** 298 45 N

**324** 243 60 N

**325** 228 55 N

**326** 240 58 N

**327** 256 52 N

**328** 270 52 N

**329** 260 52 N

**330** 280 48 N

**331** 278 40 N

**332** 260 58 N

**333** 240 40 N

**334** 248 42 N

**335** 254 46 N

**336** 276 48 N

**337** 255 48 N

**338** 258 46 N

**339** 250 45 N

**340** 245 50 N

**341** 230 50 N

**342** 234 46 N

**343** 258 40 N

**344** 250 68 N

**345** 252 54 N

**346** 234 58 N

**347** 275 60 N

**348** 270 64 N

**349** 275 66 N

**350** 296 50 N

**351** 310 42 N

**352** 263 36 N

**353** 264 48 N

**354** 268 48 N

**355** 290 38 N

**356** 284 44 N

**357** 280 42 N

**358** 274 38 N

**359** 284 45 N

**360** 278 44 N

**361** 275 40 N

**362** 270 42 N

**363** 248 32 W

**364** 252 32 W

**365** 264 60 W

**366** 235 65 W

**367** 255 49 W

**368** 255 46 W

**369** 108 36 W

**370** 260 38 N

**371** 266 36 W

**372** 240 60 N

**373** 310 65 N

**374** 333 76 N

**375** 226 58 N

**376** 304 60 N

**377** 305 58 N

**378** 315 60 N

**379** 305 54 N

**380** 295 46 N

**381** 285 48 N

**382** 260 45 N

**383** 245 42 N

**384** 290 72 N

**385** 292 55 N

**386** 292 62 N

**387** 285 72 N

**388** 300 62 N

**389** 316 68 N

**390** 320 84 N

**391** 144 90

**392** 296 70 N

**393** 285 60 N

**394** 270 55 N

**395** 262 64 N

**396** 260 55 N

**397** 260 54 N

**398** 280 48 N

**LINEATIONS**

| **N°** | **Trend** | **Plunge** |
| --- | --- | --- |

**1** 308 50

**2** 278 28

**3** 244 51

**4** 324 50

**5** 020 33

**6** 32 40

**7** 298 56

**8** 292 38

**9** 107 43

**10** 288 54

**11** 312 64

**12** 328 69

**13** 308 52

**14** 312 42

**15** 236 39

**16** 290 43

**17** 286 41

**18** 310 28

**19** 32 38

**20** 306 39

**21** 298 42

**22** 278 40

**23** 286 40

**24** 294 51

**25** 288 25

**26** 262 44

**27** 320 62

**28** 310 40

**29** 310 38

**30** 336 25

**31** 000 49

**32** 210 79

**33** 350 48

**34** 345 40
